# Identifying misbonded atoms in the 2019 CoRE metal–organic framework database†

**DOI:** 10.1039/d0ra02498h

**Published:** 2020-07-20

**Authors:** Taoyi Chen, Thomas A. Manz

**Affiliations:** Department of Chemical & Materials Engineering, New Mexico State University Las Cruces New Mexico 88003-8001 USA tmanz@nmsu.edu

## Abstract

Databases of experimentally-derived metal–organic framework (MOF) crystal structures are useful for large-scale computational screening to identify which MOFs are best-suited for particular applications. However, these crystal structures must be cleaned to identify and/or correct various artifacts. The recently published 2019 CoRE MOF database (Chung *et al.*, *J. Chem. Eng. Data*, 2019, **64**, 5985–5998) reported thousands of experimentally-derived crystal structures that were partially cleaned to remove solvent molecules, to identify hundreds of disordered structures (approximately thirty of those were corrected), and to manually correct approximately 100 structures (*e.g.*, adding missing hydrogen atoms). Herein, further cleaning of the 2019 CoRE MOF database is performed to identify structures with misbonded or isolated atoms: (i) structures containing an isolated atom, (ii) structures containing atoms too close together (*i.e.*, overlapping atoms), (iii) structures containing a misplaced hydrogen atom, (iv) structures containing an under-bonded carbon atom (which might be caused by missing hydrogen atoms), and (v) structures containing an over-bonded carbon atom. This study should not be viewed as the final cleaning of this database, but rather as progress along the way towards the goal of someday achieving a completely cleaned set of experimentally-derived MOF crystal structures. We performed atom typing for all of the accepted structures to identify those structures that can be parameterized by previously reported forcefield precursors (Chen and Manz, *RSC Adv*., 2019, **9**, 36492–36507). We report several forcefield precursors (*e.g.*, net atomic charges, atom-in-material polarizabilities, atom-in-material dispersion coefficients, electron cloud parameters, *etc.*) for more than five thousand MOFs in the 2019 CoRE MOF database.

## Abstract 2

The 2019 CoRE MOF database was cleaned by identifying structures containing isolated atoms, overlapping atoms, misplaced hydrogens, and under/over-bonded carbons.

## Graphical abstract

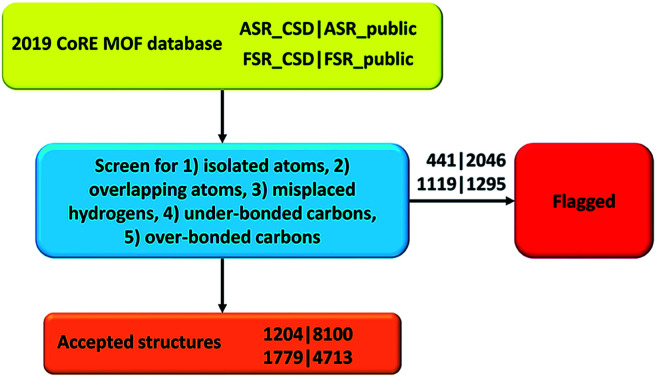

## Introduction

Metal–organic frameworks (MOFs) contain organic ligands connected by metal atoms to form coordination networks.^1–5^ MOFs that are nanoporous crystals attract much interest for gas storage, gas separation, catalysis, and other applications.^6–10^

In 2014, Chung *et al.*^11^ reported a Computation Ready Experimental (CoRE) MOF database that was constructed by first searching the Cambridge Structural Database^12,13^ (CSD) to identify MOFs and then partially cleaning these structures. Their cleaning procedure intended to remove solvent molecules and other small adsorbates in the MOF's pores, to retain charge-balancing ions, and to fix or discard structures containing disordered atoms and partial occupancies.^11^ Missing hydrogen atoms were added to some of the structures. However, this cleaning process was imperfect resulting in some structures with errors.^14–17^ Whether or not these structural errors are fixed can impact gas adsorption properties.^18^

Our previous study performed quantum chemistry calculations on the majority of structures in the 2014 CoRE MOF database.^17^ We screened out 1501 structures that contained isolated atom(s) or gave unreliable results: negative charges on metal atoms, sum of bond orders (SBOs) that were too high or too low, or large errors in the electrostatic potential model. We reported forcefield precursor parameters including net atomic charges, London dispersion coefficients, atom-in-material polarizabilities, *etc.* for 3056 out of 5109 MOFs. We also introduced a second-neighbor-based atom typing scheme and reported average forcefield precursor values for each atom type.

**Fig. 1:**
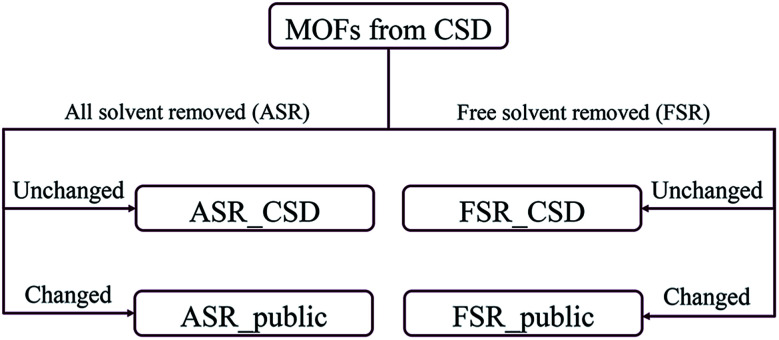
Flow diagram for the construction of CoRE MOF 2019 database.

Recently, Chung *et al.* reported an updated version of the database, CoRE MOF 2019, that includes several thousand more structures.^19^ Starting structures were put through two solvent removal procedures. The free solvent removed (FSR) set contains structures with only free solvent molecules removed. The all solvent removed (ASR) set contains structures with both free and bound solvent molecules removed. In cases where the FSR or ASR procedures did not result in any removed molecules, Chung *et al.* reported the original CSD refcode as the relevant structure. This divided the CoRE MOF 2019 database into four subsets: ASR_CSD and FSR_CSD for CSD structures that were unmodified when the ASR or FSR cleaning procedure was applied, and ASR_public and FSR_public for structures that were modified during the ASR or FSR cleaning procedure, respectively. Fig. 1 shows how the CoRE MOF 2019 database is constructed and divided into four subsets. They also pointed out that the ASR set and the CoRE MOF 2014 database underwent similar solvent removal procedures; 5009 of 5109 MOFs from the CoRE MOF 2014 database are in the CoRE MOF 2019 ASR dataset.^11,19^

**Fig. 2:**
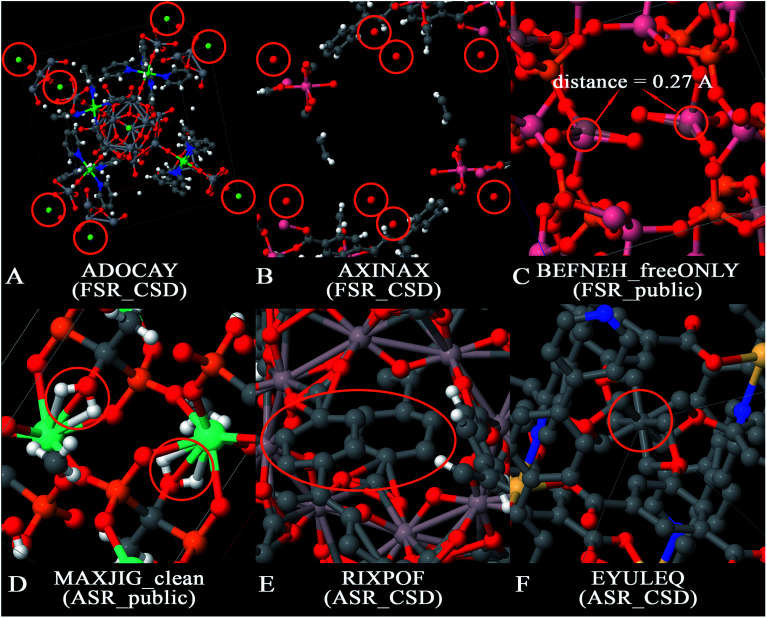
Examples of artifacts being screened in this paper. Panel (A) is an example of isolated atoms in the data that are likewise isolated in the real physical specimen (the circled atoms are F^−^ ions). Panel (B) is an example of isolated atoms in the data that are likely not isolated in the real physical specimen (the circled atoms are oxygen atoms which likely belong to water molecules in the physical specimen for which hydrogen atoms were omitted in the reported crystal structure). Panel (C) is an example of overlapping atoms. Panel (D) is an example of misplaced hydrogens. Panel (E) is an example of under-bonded carbons. Panel (F) is an example of over-bonded carbons.

There are several opportunities to further clean the CoRE MOF 2019 dataset. For example, Chung *et al.* identified disordered structures as those having atoms closer than 0.1 Å (*i.e.*, overlapping atoms).^19^ Because the H_2_ molecule's bond length of 0.74 Å is one of the shortest bond lengths in chemistry, the criterion for overlapping atoms could be made less strict than atoms ≤ 0.1 Å apart. There is also a need to identify missing or misbonded hydrogen atoms and isolated atoms. In this paper, we cleaned the database from the following aspects: (1) isolated atoms (*i.e.*, atoms or atomic ions not directly bonded to any neighboring atoms), (2) atoms too close together (*i.e.*, overlapping atoms), (3) misplaced hydrogen atoms, (4) under-bonded carbon atoms (which might be due to missing hydrogen atoms), and (5) over-bonded carbon atoms. Fig. 2 shows example MOFs for each artifact being screened in this study.

The term artifacts has the following meaning. First, the term artifact refers to a property of the data rather than a property of the material itself. (Here, the term “material itself” refers to a physical specimen of the material.) For example, X-ray crystallography of a physical specimen containing disordered atoms or twinned crystal structures often yields data (*i.e.*, reported crystal structure geometry) exhibiting overlapping atoms; no overlapping atoms exist in the physical specimen. In this article, the term ‘overlapping atoms’ means atoms that are much too close together. Missing hydrogen atoms is another artifact: the data (*i.e.*, reported crystal structure geometry) is often missing one or more hydrogen atoms, but no hydrogen atoms are missing in the physical specimen of the material. Under-bonded carbon atoms may be caused by missing hydrogen atom(s) in the data; these are normally not under-bonded in the physical specimen. Over-bonded carbon atoms may be caused by overlapping atoms; these are normally not over-bonded in the physical specimen. In this article, the term ‘isolated atom’ does not mean a single atom in empty space, but rather an atom that is not covalently bonded to any neighboring atoms and hence may be labile to easy replacement (*e.g.*, ion exchange). Two different scenarios arise for the isolated atoms. The first scenario corresponds to an isolated atom in both the data and the physical specimen. Fig. 2A shows an example in which a MOF contains isolated F^−^ ions; these ions might be exchangeable for Cl^−^ or other ions if the MOF is placed in solution. Instead of anions, physical specimens might also contain isolated cations (*e.g.*, Na^+^, Sr^+*q*^, *etc.*) or potentially even an isolated neutral atom. The potential for anion or cation exchange makes it worthwhile to flag these structures. The second scenario corresponds to an isolated atom in the data that is not an isolated atom in the physical specimen. Fig. 2B shows an example in which a reported MOF structure contains isolated O atoms, but these are almost certainly water molecules in the physical specimen for which the hydrogen atoms were not included in the reported crystal structure geometry.

Here, we have flagged rather than deleted structures containing these artifacts. Flagging the structures, rather than deleting them, will make it easier for those structures to be corrected in future work without having to re-insert them into the database. Specifically, any structure corrected in future work could have a new flag added that links to the corrected structure. Also, flagging these artifacts provides flexibility in how the database is used for computational screening studies. Depending on the target application, database users may want to include or exclude various categories of the flagged structures.

As its name indicates, the Computational Ready Experimental (CoRE) MOF database was created for the purpose of providing a library of MOF crystal structures in a format ready to be used as input for large-scale computational screening studies (*e.g.*, classical molecular dynamics or Monte Carlo simulations for gas separation applications).^11^ Geometries with misbonded atoms (*e.g.*, overlapping atoms, misplaced hydrogen atoms, under-bonded carbons, over-bonded carbons) are not in a format ready to perform classical molecular dynamics or Monte Carlo simulations; hence, the reason for flagging those structures. We also chose to flag structures containing isolated atoms to allow users the ability to choose whether or not to include those structures in their computational screening studies. In some cases, isolated atoms exist in the real physical specimen (*e.g.*, F^−^, Cl^−^, Na^+^, *etc.*) while in other cases it is an error of the crystal structure refinement procedure (*e.g.*, an isolated O atom in the data that corresponds to a water molecule in the physical specimen for which the H atoms were omitted during crystal structure refinement).

Another opportunity is to perform atom typing and to assign forcefield precursors to the CoRE MOF 2019 structures. After screening for misbonded or isolated atoms, we performed second-neighbor-based atom typing on all accepted structures from the CoRE MOF 2019 dataset. Several forcefield precursors were then assigned to those structures that contained previously parameterized^17^ atom types. Atom types simplify forcefield parameterization. Sufficiently similar atoms are classified as the same atom type. Atoms of the same type are normally assigned the same forcefield precursor values. Forcefield precursors are building blocks that are combined to construct a force field.^20^ For example, electrostatic models can be constructed using the net atomic charges^21^ and/or atomic multipoles and/or polarizabilities and/or electron cloud (charge penetration) parameters. Dispersion models can be constructed using the C_6_, C_8_, and/or C_10_ dispersion coefficients and/or the quantum Drude oscillator parameters. (The C_9_ dispersion coefficients can also be computed from these forcefield precursors.^22^) Protocols have to be developed and tested for turning these forcefield precursors into working force fields for MOFs. Simpler forcefield forms, such as Lennard-Jones parameters, can potentially be derived from these forcefield precursors. (Cole *et al.*^23–26^ and Nikitin^27^ introduced methods to compute Lennard-Jones parameters for small molecules and large biomolecules from DDEC atom-in-material descriptors, and they used these in classical atomistic simulations.)

## Methods

Our analyses for misbonded atoms used the atom typing radii (ATR) reported in our previous study.^17^ Our atom typing radii are intended to be effective atomic radii in the typical charge state of the atom in materials. We assigned a bond between two atoms if and only if the distance between them was less than or equal to the sum of their ATR. In our prior work, we optimized these ATR through trial and error (starting from the Open Babel version 1.100.1 connectivity radii as initial guesses) to produce reasonable connectivity results for various MOFs.^17^ Covalent radii are designed to be effective atomic radii in covalent single bonds.^28^ In MOFs, metal atoms typically carry positive atomic charges, so the effective atomic radii of metal atoms in MOFs are not necessarily similar to their covalent radii. Specifically, our ATR of metal atoms are often somewhat smaller than their covalent radii. We found this greatly improves connectivity results compared to using covalent radii for atom typing, because using covalent radii for atom typing often yields unreasonably high coordination numbers for metal atoms.

The screening was performed on all four subsets: ASR_CSD, ASR_public, FSR_CSD, and FSR_public. An atom was considered isolated if it was not connected to any other atom based on the ATR. Two atoms were considered overlapping if the distance between them was smaller than half the sum of their ATR.

Misplaced hydrogen atoms were identified using the following procedure. For each hydrogen atom, a list was constructed containing atoms located within a distance equal to the sum of ATR plus 0.3 Å. If the list for one hydrogen atom contained at least one metal atom and one oxygen or nitrogen atom, this hydrogen atom was considered misplaced. The rational for this is if a hydrogen atom is bonded to a nitrogen or oxygen, the hydrogen atom will be more positively charged than usual and repelled by positively charged metal atoms. In contrast, hydrogen atoms bonded to carbon are known to be able to participate in agostic bonds (*i.e.*, C–H–metal bonds).^29^

To screen out structures with under-bonded and/or over-bonded carbon atoms, we performed an empirical carbon bond order analysis. We chose a purely distance-based calculation of bond orders, because misbonded atoms (*e.g.*, overlapping atoms or missing hydrogens) make it unreliable to infer bond orders from connectivity patterns alone. We collected the carbon DDEC6 bond order^30^*versus* bond length information from our previously published 3056 forcefield precursor (FFP) MOFs.^17^ The data were fit to the following equation

1 log_10_(BO) = *A* × *d* + *C*

where log_10_ is the base 10 logarithm, BO is the bond order, *A* is the slope, *d* is the distance between two atoms, and *C* is a constant. This relation was first proposed by Pauling in 1947.^31^ Element pairs without sufficient or diverse data to provide a meaningful fit were excluded. Table 1 lists the coefficients and goodness of fit for eqn (1) for C–H, C–B, C–C, C–N, C–O, C–Cl, and C–Br pairs. The raw data is found in ESI Part S01.† The DDEC6 bond order is defined such that the dressed self-exchange B_AA_ for atom A is no less than half the self-contact exchange CE_AA_.^30^ Because hydrogen atoms have no core electrons, this constraint is often binding for hydrogen atoms and almost never binding for heavier elements.^30^ Accordingly, the empirical C–H bond order was constrained using the equation

2 BO_C–H_ = min(1.25,−0.6093 × *d* + 0.5927)

where 1.25 represents an allowed upper bound on the C–H bond order. Examining Table 1, the slope for C–B was substantially higher in magnitude than for C–C or C–N; this appears to be due to a more limited amount of C–B fitting data compared to C–C and C–N. Therefore, the C–B correlation should not be extrapolated far beyond the range of C–B distances for which it was fit.

**Table:** Coefficients for eqn (1) for fitted C-atom bond orders

Atom	*A* (Å^−1^)	*C*	*R* ^2^
H	−0.6093	0.5927	0.7584
B	−2.2011	3.4380	0.9638
C	−1.2685	1.8855	0.9233
N	−1.2680	1.8401	0.9255
O	−1.0525	1.5189	0.9477
Cl	−0.7621	1.3723	0.9350
Br	−0.8003	1.5272	0.9776

Because carbon has four electrons to share in covalent bonding, the sum of bond orders (SBO) is expected to be approximately four for each carbon atom in most organic and organometallic compounds. The sum of ATR was used to identify all atoms directly bound to each carbon atom. If a carbon atom was bound only to the elements listed in Table 1, and its empirical SBO (computed using the parameters in Table 1) was smaller than 3.3, the structure containing that carbon atom was flagged for under-bonded carbon atom; the structure was flagged for over-bonded carbon atom if the SBO was greater than or equal to 5.5. These empirical SBO thresholds of 3.3 and 5.5 for carbon atoms were set more generous than the DDEC6 SBO thresholds of 3.5 and 4.75 used in our previous study^17^ to account for the larger chemical uncertainty associated with the empirical SBO value compared to the quantum-mechanically computed DDEC6 SBO value. This wider threshold increases the tolerance for how much a computed carbon SBO could differ from ∼4 before the structure was flagged.

This procedure can screen out structures missing hydrogen atoms on carbon atoms connected only to H, B, C, N, O, Cl, and/or Br atoms. For example, a carbon atom missing a hydrogen atom might have a computed SBO value of ∼3 instead of ∼4. A carbon atom missing two hydrogen atoms might have a computed SBO value of ∼2 instead of ∼4. Notably, this procedure does not screen carbon atoms connected to other elements (*e.g.*, metal atoms) for missing hydrogen atoms. Therefore, more sophisticated screening strategies may be required in future work to identify all structures missing hydrogen atoms. Our goal here was to perform screening that could reliably improve the database by identifying some structures missing hydrogen atoms, even if that screening did not identify all structures missing hydrogen atoms.

A pseudocode for screening out (1) isolated atoms, (2) overlapping atoms, (3) misplaced hydrogens, (4) under-bonded carbons and (5) over-bonded carbons is in ESI Part S17.† A Python function that performs the second-neighbor-based atom typing is in ESI Part S18.† Of course, both the pseudocode of ESI Part S17† and the Python atom typing function of ESI Part S18† look across the periodic boundary conditions to identify all the relevant neighbors of atoms in the reference unit cell, even if some of these neighbors are in adjacent unit cells.

## Results and discussion

In the CoRE MOF 2019 database, Chung *et al.* labeled structures with the distance between two atoms ≤ 0.1 Å as disordered.^19^ They also manually moved some structures into the disordered category based on user feedback (see DOI: 10.5281/zenodo.3528250). Because disordered atoms make these structures unsuitable for classical atomistic or quantum-mechanical simulations, all of those disordered structures were not included in our present study. The all solvent removal (ASR) criterion is more stringent than the free solvent removal (FSR) criterion.^19^ This has two implications. First, all structures modified by the FSR procedure should also be modified by the ASR procedure. Therefore, we systematically checked for structures violating this rule and found three: NODTEH, NODTIL and NODTOR. These three were removed from ASR_CSD and added to ASR_public using their FSR_public geometries. Second, all structures unmodified by ASR should also remain unmodified by FSR. Therefore, we added 278 ASR_CSD structures that were not in FSR_CSD to FSR_CSD before the screening. The detailed list is in ESI Part S04.† We removed some structures from the database because their parent structure no longer exists in the CSD database; the list of such structures is in ESI Part S02.†

Tables 2 and 3 list the breakdown of flagged structures due to the five major artifacts. Structures not flagged with any of these five artifacts were marked as ‘accepted’. The numbers for each flag criterion do not add up to the total number because of the overlap between categories. The detailed lists of artifacts in structures for each subset are in ESI Part S03.† As summarized in Table 4 and listed in ESI Part S03,† we also searched for structures that did not contain any hydrogen atoms or carbon atoms. Technically, the structures not containing carbon atoms should be referred to as metal–inorganic frameworks (MIFs) rather than as MOFs.^15,17,32,33^

**Table:** Breakdown of flagged MOFs of major artifacts from each subset. The number of structures containing only that artifact type is listed in parentheses

	Isolated atoms	Misbonded hydrogens	Overlapping atoms	Under-bonded carbons	Over-bonded carbons	Total flagged	Accepted
ASR_CSD	88 (72)	20 (16)	100 (33)	201 (154)	137 (70)	441	1204
ASR_public	819 (718)	132 (107)	127 (93)	1041 (922)	91 (51)	2046	8100
FSR_CSD	218 (149)	44 (28)	445 (101)	433 (281)	481 (127)	1119	1779
FSR_public	485 (405)	82 (63)	70 (46)	727 (629)	63 (29)	1295	4713

Number of structures containing two or more types of artifacts in isolated atoms (IA), overlapping atoms (OA), misbonded hydrogens (MH), under-bonded carbons (UC), or over-bonded carbons (OC). The listed number is for those structures containing only the listed artifacts (*e.g.*, structures listed under IA/OA/UC are not included in structures listed under IA/OA)

**Table:** 

	IA/MH	IA/OA	IA/UC	IA/OC	MH/OA	MH/UC	MH/OC
ASR_CSD	1	5	7	0	0	1	1
ASR_public	5	12	78	1	3	6	7
FSR_CSD	2	11	30	1	4	3	1
FSR_public	2	8	62	1	3	5	7

**Table:** 

	OA/UC	OA/OC	UC/OC	IA/MH/OA	IA/MH/UC	IA/MH/OC	IA/OA/UC
ASR_CSD	12	41	19	0	0	0	2
ASR_public	7	6	21	1	0	1	1
FSR_CSD	21	243	40	0	0	0	5
FSR_public	5	3	18	1	1	0	2

**Table:** 

	IA/OA/OC	IA/UC/OC	MH/OA/UC	MH/OA/OC	MH/UC/OC	OA/UC/OC	IA/MH/OA/UC
ASR_CSD	1	0	1	0	0	5	0
ASR_public	0	2	2	0	0	2	0
FSR_CSD	16	1	3	3	0	46	0
FSR_public	0	3	0	0	0	2	0

**Table:** 

	IA/MH/OA/OC	IA/MH/UC/OC	IA/OA/UC/OC	MH/OA/UC/OC	All 5
ASR_CSD	0	0	0	0	0
ASR_public	0	0	0	0	0
FSR_CSD	0	0	3	0	0
FSR_public	0	0	0	0	0

**Table:** Number of structures not containing hydrogen or carbon atoms

	Total structures	No hydrogens	No carbons
ASR_CSD	1645	48	9
ASR_public	10 146	859	463
FSR_CSD	2898	74	10
FSR_public	6008	473	300

In our previous study, we reported 7033 second-neighbor-based atom types for the FFP MOFs with their forcefield precursor parameters.^17^ The standard deviation of calculated forcefield precursor values was relatively small across atoms sharing the same second neighbor environments.^17^ ESI Parts S05–S08† list second-neighbor-based atom types contained in each structure for the accepted_ASR_CSD, accepted_ASR_public, accepted_FSR_CSD, and accepted_FSR_public sets. ESI Part S09† lists the frequencies for all atom types in these subsets. 3274 different atom types were found in the accepted_ASR_CSD structures, 14 710 in accepted_ASR_public structures, 4911 in accepted_FSR_CSD structures, and 11 175 in accepted_FSR_public structures. This clearly demonstrates high chemical diversity in the 2019 CoRE MOF database. ESI Parts S10 and S11† list the *XYZ* coordinates and atom type for each atom in the accepted_ASR_public and accepted_FSR_public structures. *XYZ* coordinates for the CSD structures must be obtained through the CSD.^12,13^

In general, two crystal structures could be considered to be chemically equivalent if all of the following criteria are met:

(1) The two structures contain the same chemical elements.

(2) The number of atoms of each chemical element divided by the unit cell volume is the same for both structures. This criterion identifies a non-interpenetrating MOF and an interpenetrating version of this MOF as distinct structures; in this case, the interpenetrating MOF would have twice the number of atoms of each chemical element per unit cell volume compared to the non-interpenetrating MOF.^15^

(3) The two structures have similar geometric conformations. Rotational and translational invariance must be considered when evaluating this criterion. This criterion distinguishes two MOFs having similar chemical elements arranged in different chemical conformations. For example, two different geometric isomers, enantiomers (optical isomers), or other conformations would be considered different structures.

(4) The two structures have the same crystal polymorph.

Here, we are interested in the more restricted question of whether two structures having the same reference code but appearing in two different datasets are equivalent. Two structures having the same reference code were derived from the same experimental crystal structure (*i.e.*, same physical specimen) using different cleaning protocols. Because these structures were derived from the same experimental crystal structure, criteria (3) and (4) are necessarily satisfied if criteria (1) and (2) are satisfied. Therefore, an ASR_public structure with reference code (*e.g.*, XXXXXX_clean) was considered equivalent to a corresponding FSR_public structure having analogous reference code (*e.g.*, XXXXXX_freeONLY) if and only if criteria (1) and (2) above are satisfied. Two reference codes were considered to be analogous if they had the same journal-based code or six-digit CSD code, irrespective of the added CoRE MOF suffix (*e.g.*, _clean, _freeONLY). Therefore, two structures of different subsets having the same reference code were considered equivalent if they satisfied criteria (1) and (2) above. We did not screen for whether two structures having different reference codes (*i.e.*, derived from two different physical specimens) were equivalent. We found 3924 structures shared between the ASR_public and FSR_public subsets, 2606 structures shared between the ASR_public and FFP^17^ sets, and 1054 structures shared between FSR_public subset and FFP sets. These shared structures represent cases for which two different cleaning procedures (*i.e.*, ASR, FSR, CoRE2014) produced identical ‘cleaned’ structures derived from the same physical specimen. We report the codes for these shared structures in ESI Part S12.†

In contrast, ESI Part S13† lists composition differences between ASR_public and FSR_public structures that have the same reference codes but different chemical compositions. These structures do not satisfy criterion (1) and/or (2) above. These are cases for which the FSR cleaning procedure produced a substantially different result than the ASR cleaning procedure applied to the experimental crystal structure of the same physical specimen.

ESI Part S14† lists the 700 accepted_ASR_CSD, 4701 accepted_ASR_public, 716 accepted_FSR_CSD, and 1904 accepted_FSR_public structures that can be fully described by the 7033 atom types for which we previously reported^17^ forcefield precursor values. These structures are computational ready for forcefield simulations using our reported atom type forcefield precursor parameters. ESI Parts S15 and S16† list the *XYZ* coordinates together with the following forcefield precursor values for every atom in accepted_ASR_public and accepted_FSR_public structures that can be fully described by the reported atom types: net atomic charge;^34,35^ C_6_, C_8_, and C_10_ dispersion coefficients;^22,36^ three kinds of polarizabilities (*i.e.*, fluctuating, isotropic forcefield, and static);^22,36^ parameters fitting the atom's electron density tail to an exponential function (*i.e.*, electron cloud parameters);^17^ 〈*r*^3^〉 and 〈*r*^4^〉 radial moments; quantum Drude oscillator parameters;^22,36^ and atomic dipole magnitude. The atomic spin moment is not included here among the forcefield precursors, because magnetic ordering is almost energy degenerate (and hence hard to accurately predict) in some materials.^37,38^

The net atomic charges in these structures were rescaled to make the overall unit cell charge equal zero. If the unit cell charge before rescaling was >0, then only the NACs > 0 were proportionally rescaled to make the rescaled unit cell charge zero. If the unit cell charge before rescaling was <0, then only the NACs < 0 were proportionally rescaled to make the rescaled unit cell charge zero. This conservative rescaling changes the NAC magnitudes by the smallest percentage possible to achieve unit cell neutrality while never increasing the NAC magnitude for any atom. Because the root-mean-squared error (RMSE) of the electrostatic potential is more sensitive to large magnitude NACs than to small magnitude NACs, we chose not to increase NAC magnitudes during rescaling.

These forcefield precursors reported for 5000+ MOFs could be used in future work to construct working interaction models for MOFs. The simplest useful force field would consist of Lennard-Jones parameters plus the atomic charges to describe short-range repulsive interactions, long-range dispersion interactions, and electrostatic interactions between atoms in the material. A flexible force field would also require bonded atom parameters such as bond springs, angle springs, and torsion parameters. The Manz research group is currently in the process of developing and testing short-range repulsion formulas that are computed from the electron cloud parameters reported herein as force field precursors. We are also using this short-range repulsion function as the basis to construct the argument for Tang–Toennies damping^39,40^ of the C_6_, C_8_, and C_10_ dispersion terms reported herein. Finally, the Manz research group is currently testing this short-range repulsion together with damped dispersion and intends to publish a follow-up article that will describe how to turn these forcefield precursors into working interaction models.

## Conclusion

**Fig. 3:**
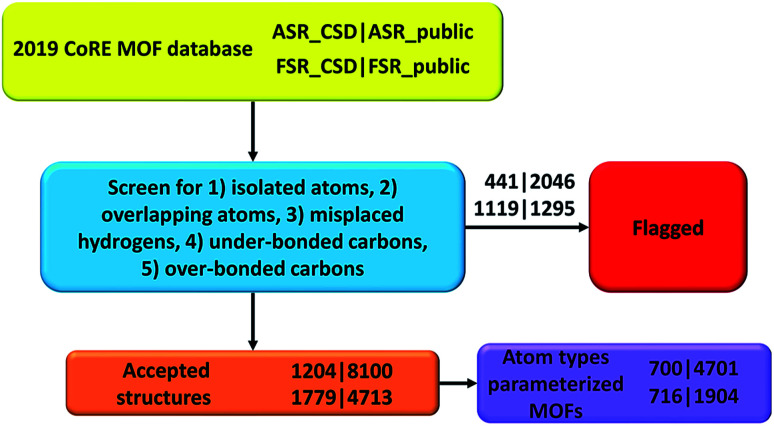
Flow diagram of this project.

In this paper, we screened the 2019 CoRE MOF database to flag structures containing isolated or misbonded atoms: (i) atoms not directly bonded to any neighboring atoms (*i.e.*, ‘isolated’ atoms), (ii) atoms too close together (*i.e.*, overlapping atoms), (iii) misplaced hydrogen atoms, (iv) under-bonded carbon atoms (which might be caused by missing hydrogen atoms), and (v) over-bonded carbon atoms. Depending on the situation, an ‘isolated’ atom may correspond to an exchangeable atom (*e.g.*, F^−^, Cl^−^, Na^+^, Sr^+*q*^) or an error of the crystal structure refinement procedure (*e.g.*, a water molecule whose hydrogen atoms were not reported could appear as an isolated oxygen atom). This study should not be viewed as the final cleaning of this database, but rather as progress along the way towards the goal of someday achieving a completely cleaned set of experimentally-derived MOF crystal structures. This resulted in the following numbers of accepted structures: 1204 in accepted_ASR_CSD, 8100 in accepted_ASR_public, 1779 in accepted_FSR_CSD, and 4713 in accepted_FSR_public. We performed several kinds of comparative analysis: (a) structures not containing hydrogen or carbon atoms, (b) structures common to two or more of the datasets, and (c) composition differences between ASR_public and FSR_public structures having the same reference codes. We performed atom typing for all of the accepted structures. We identified 700 of 1204 accepted_ASR_CSD, 4701 of 8100 accepted_ASR_public, 716 of 1779 accepted_FSR_CSD, and 1904 of 4713 accepted_FSR_public structures that can be parameterized by our previously reported^17^ forcefield precursors. For accepted_ASR_public and accepted_FSR_public structures that can be described by the reported atom types, the following forcefield precursors are listed for each atom: net atomic charge; C_6_, C_8_, and C_10_ dispersion coefficients; three kinds of polarizabilities (*i.e.*, fluctuating, isotropic forcefield, and static); parameters fitting the atom's electron density tail to an exponential function (*i.e.*, electron cloud parameters); 〈*r*^3^〉 and 〈*r*^4^〉 radial moments; quantum Drude oscillator parameters; and atomic dipole magnitude. The procedures and results are summarized in Fig. 3. In summary, our results facilitate future computational screening studies of MOFs by making this database cleaner and by providing atom types and forcefield precursors. Future work will address the task of turning these forcefield precursors into working force fields.

## Authors' contributions

T. C. performed the computational screening and data analysis. Both authors designed the study and wrote the manuscript.

## Conflicts of interest

There are no conflicts of interest to declare.

## Supplementary Material

RA-010-D0RA02498H-s001

RA-010-D0RA02498H-s002
